# Administration of the NOD2 Agonist MDP Reduces *Cryptosporidium parvum* Infection in Neonatal Mice Through IL‐22 Involvement

**DOI:** 10.1002/eji.70080

**Published:** 2025-10-20

**Authors:** Mégane Fernandez, Tiffany Pezier, Julien Pichon, Yves Le Vern, Catherine Werts, Sonia Lacroix‐Lamandé

**Affiliations:** ^1^ INRAE, Université de Tours, UMR 1282 Infectiologie et Santé Publique, Laboratoire AIM Nouzilly France; ^2^ INRAE, Université de Tours, UMR 1282 Infectiologie et Santé Publique, Laboratoire IMI Nouzilly France; ^3^ Institut Pasteur, INSERM U1306, Unité de Biologie et Génétique de La Paroi Bactérienne Paris France

**Keywords:** *Cryptosporidium parvum*, IL‐22, intestine, neonates, nucleotide‐binding oligomerization domain 2 (NOD2)

## Abstract

At birth, the mucosal immune system of neonates is not fully developed, making them more susceptible to respiratory and intestinal diseases. Previously described host‐directed therapies using toll‐like receptor (TLR) activation‐based strategies have proven effective in controlling neonatal diseases, including cryptosporidiosis. In this study, we investigated the effect of nucleotide‐binding oligomerization domain (NOD) receptors stimulation on the control of enteric infection by the protozoan *Cryptosporidium parvum* in neonatal mice. NOD2 stimulation by intraperitoneal injection of muramyl dipeptide (MDP) resulted in a rapid reduction in the parasite burden. The protective effect was associated with increased pro‐inflammatory cytokine and antimicrobial peptide gene expression and a rapid influx of neutrophils to the site of infection, whereas NOD1 stimulation did not show a protective effect. The protective mechanism did not involve microbiota participation but involved IFN‐γ and IL‐22 cytokines and was associated with increased intestinal epithelium renewal in infected neonates. Our findings showed that stimulating neonatal mice with the bacterial ligand MDP, which targets the NOD2 receptor, actively contributes to the nonspecific clearance of *C. parvum* infection by eliminating or renewing infected epithelial cells.

## Graphical abstract

Neonatal mice infected with C. parvum and treated with MDP, a NOD2 agonist, exhibited reduced intestinal infection. This protective effect was mediated by a local immune response involving neutrophil recruitment, IL‐22 production, antimicrobial peptide expression, and epithelial regeneration in an IFN‐γ‐dependent manner. Therefore, MDP administration enhances host defense mechanisms against C. parvum infection in neonatal mice. Created with BioRender.com.

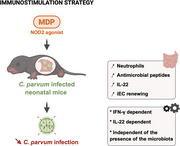

## 1 Introduction

Cryptosporidiosis is an intestinal disease caused by the protozoan *Cryptosporidium parvum* (*C. parvum*), typically transmitted through oral ingestion. This parasite develops in intestinal epithelial cells (IECs) and causes acute diarrhea in very young ruminants, young children, and immunocompromised hosts [1, 2]. The hallmark of this disease is acute gastrointestinal distress manifested by diarrhea, abdominal cramps, nausea, vomiting, and fever. In humans, the most susceptible groups include children under the age of 5 and immunocompromised individuals [2]. Importantly, *C. parvum* is the second most common cause of moderate to severe diarrhea among children under the age of 2 [3]. In contrast, in cattle, cryptosporidiosis is recognized as endemic worldwide and is one of the most important causes of neonatal enteritis in calves, resulting in substantial financial losses for livestock farmers [1].

Susceptibility to this infection is intricately linked to the host immune response, highlighting the central role of the immune system in combating this parasitic invasion. We and others have highlighted the critical role of innate immunity in controlling the acute phase of *C. parvum* infection, whereas adaptive immunity is required for definitive clearance of the infection [4, 5]. Host epithelial cells play a major role in both the multiplication of the parasite and the initiation of immune responses by recruiting inflammatory immune cells such as neutrophils and dendritic cells to the site of infection [4, 6, 7, 8]. In addition, conventional type‐1 dendritic cells (cDC1s) play a major role in this innate protective response [9], whereas inflammatory monocytes rapidly recruited to the site of infection promote the loss of intestinal barrier integrity that characterizes cryptosporidiosis [10].

With no vaccine available and limited chemotherapy, there is an urgent need to develop new methods to control cryptosporidiosis. Considering the close relationship between the susceptibility of this infection and the immune status of the host, the exploration of immune‐enhancing strategies is a promising avenue to consider. Our hypothesis is that the enhancing innate immunity with potent immunostimulants, such as pattern recognition receptor (PRR) ligands, could stimulate the production of key cytokines, that may provide defense against this enteric pathogen. In the neonatal mouse model, we have previously shown that stimulation of innate immunity by toll‐like receptor (TLR) agonists, including CpG‐ODN (TLR9 ligand) or PolyI:C (TLR3 ligand), increased the intestinal immune responses and subsequently reduce the intestinal burden of the *C. parvum* parasites [11, 12].

Another class of PRRs known as nucleotide‐binding oligomerization domain (NOD) receptors plays a critical role in the function of the innate immune system [13, 14, 15, 16]. NOD1 and NOD2 belong to this intracellular PRR family and recognize different fragments of peptidoglycan (PGN), a major component of the bacterial cell wall [17]. NOD1 recognizes γ‐d‐glutamyl‐*meso*‐diaminopimelic acid (iE‐DAP) from Gram‐negative bacteria and certain Gram‐positive bacteria, and NOD2 recognizes muramyl dipeptide (MDP), which is present in both Gram‐positive and Gram‐negative bacteria. Several genome‐wide association studies have identified NOD2 as the most prevalent susceptibility factor for the development of Crohn's disease, highlighting the importance of NOD2 in intestinal immunity [16, 18, 19]. In the intestine, NOD2 is expressed by both hematopoietic and non‐hematopoietic cells that constitute the intestinal epithelium [20, 21, 22, 23, 24]. Upon activation by MDP, NOD2 facilitates host defense mechanisms by triggering the production of cytokines, chemokines, antimicrobial peptides, and mucins [20, 25, 26, 27]. Several studies have described the protective role of NOD2 in various models of intestinal inflammation [17, 27, 28]. Furthermore, activation of NOD2 has been shown to improve clearance and reduce the severity of *Salmonella* and *Citrobacter rodentium* colitis in mice, highlighting its involvement in the regulation of the intestinal immune response [29, 30]. In addition, NOD2 activation has been shown to ameliorate intestinal bacterial infections caused by *Listeria monocytogenes*, *Shigella flexneri*, and *Helicobacter hepaticus*, primarily through the production of pro‐inflammatory mediators, antimicrobial peptides, and bacterial autophagy [17, 26, 31].

In this study, we investigated the role of NOD stimulation in the regulation of *C. parvum* infection in neonatal mice. Our findings demonstrate that the stimulation of NOD2 by the MDP agonist promotes a protective effect by enhancing the innate immune response, leading to the renewal of the epithelial barrier.

## 2 Results

### 2.1 Stimulation of NOD2 Receptor Reduce *C. parvum* Intestinal Load in Infected Neonatal Mice

**FIGURE 1:**
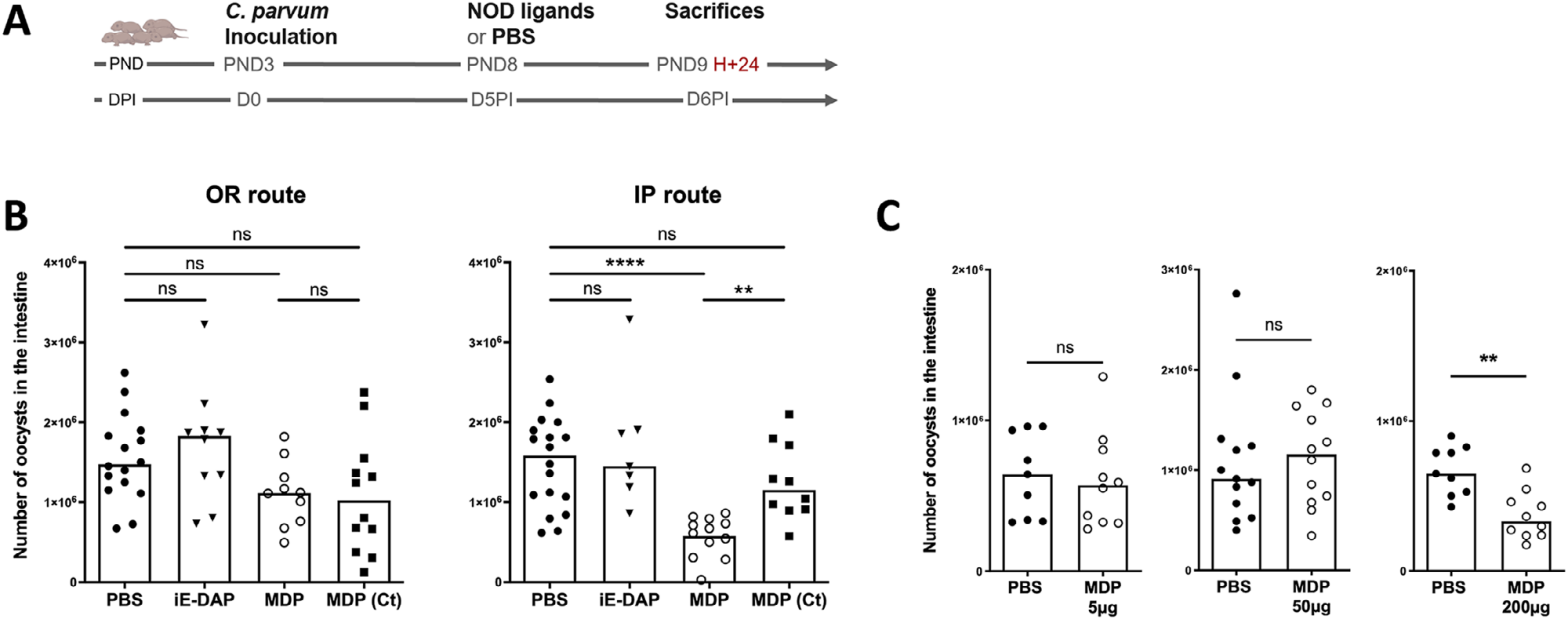
NOD2 stimulation reduces *Cryptosporidium parvum* infection in neonatal mice. (A) Experimental timeline. PND3 neonatal mice were orally infected with 5 × 10^5^ oocysts of *C. parvum* and received 200 µg of the NOD‐ligands at Day 5 p.i. (A) iE‐DAP (NOD1 ligand), MDP (NOD2 ligand) and its control MDP (MDP (Ct)), or PBS (control group) were administered by intraperitoneal (IP) or oral (OR) route to neonatal mice. (B) Parasite load in the intestine was evaluated 24 h later (*n* = 10–18 mice for each group). Statistics are calculated by the one‐way Kruskal–Wallis test followed up by Dunn's correction. (C) MDP was administered by IP route at 5, 50, and 200 µg (*n* = 9–12 mice for each group). Statistics are calculated by the Mann–Whitney test. The data are representative of three independent experiments. Each point corresponds to one mouse, and bars represent the median of each group. DPI, days post‐inoculation; IP, intraperitoneally; MDP, muramyl dipeptide; NOD, nucleotide‐binding oligomerization domain; PND, postnatal days. ns > 0.05, **p < 0.01, ****p < 0.0001.

We previously reported that stimulation of TLR9 and TLR3 receptors in neonatal mice can reduce the severity of cryptosporidiosis by enhancing a protective innate immune response in the intestine [11, 12]. Although the signal transduction pathways differ between TLR and NOD receptors upon binding of their respective agonists, we asked whether NOD receptor stimulation could elicit also a protective effect against *C. parvum* infection in neonatal mice by using the *γ*‐d‐glutamyl‐*meso*‐diaminopimelic acid (iE‐DAP) and the MDP as NOD1 and NOD2 agonists, respectively. To investigate this, neonatal mice of 3‐day‐old were inoculated orally with 5 × 10^5^ oocysts of *C. parvum* and 5 days later received 200 µg of NOD agonists orally or intraperitoneally (IP) (Figure 1A). Mice were sacrificed the following day to assess the intestinal parasite burden.

As shown in Figure 1B, no reduction in parasite load was observed after oral NOD1 or NOD2 stimulation. In contrast, IP administration of NOD2 agonist (MDP) significantly reduced the parasite load in infected neonatal mice, whereas NOD1 stimulation (iE‐DAP) was not effective. Notably, a high dose of the NOD2 ligand was required for this protection, as 5 and 50 µg of MDP administered under similar conditions did not decrease infection levels (Figure 1C). Furthermore, this protective effect was no longer observed 48 or 72 h after MDP injection, suggesting a rapid and transient impact of NOD2 stimulation in neonatal mice (Figure S1).

### 2.2 Increased Systemic and Intestinal Inflammatory Responses in Neonatal Mice IP Injected With MDP

**FIGURE 2:**
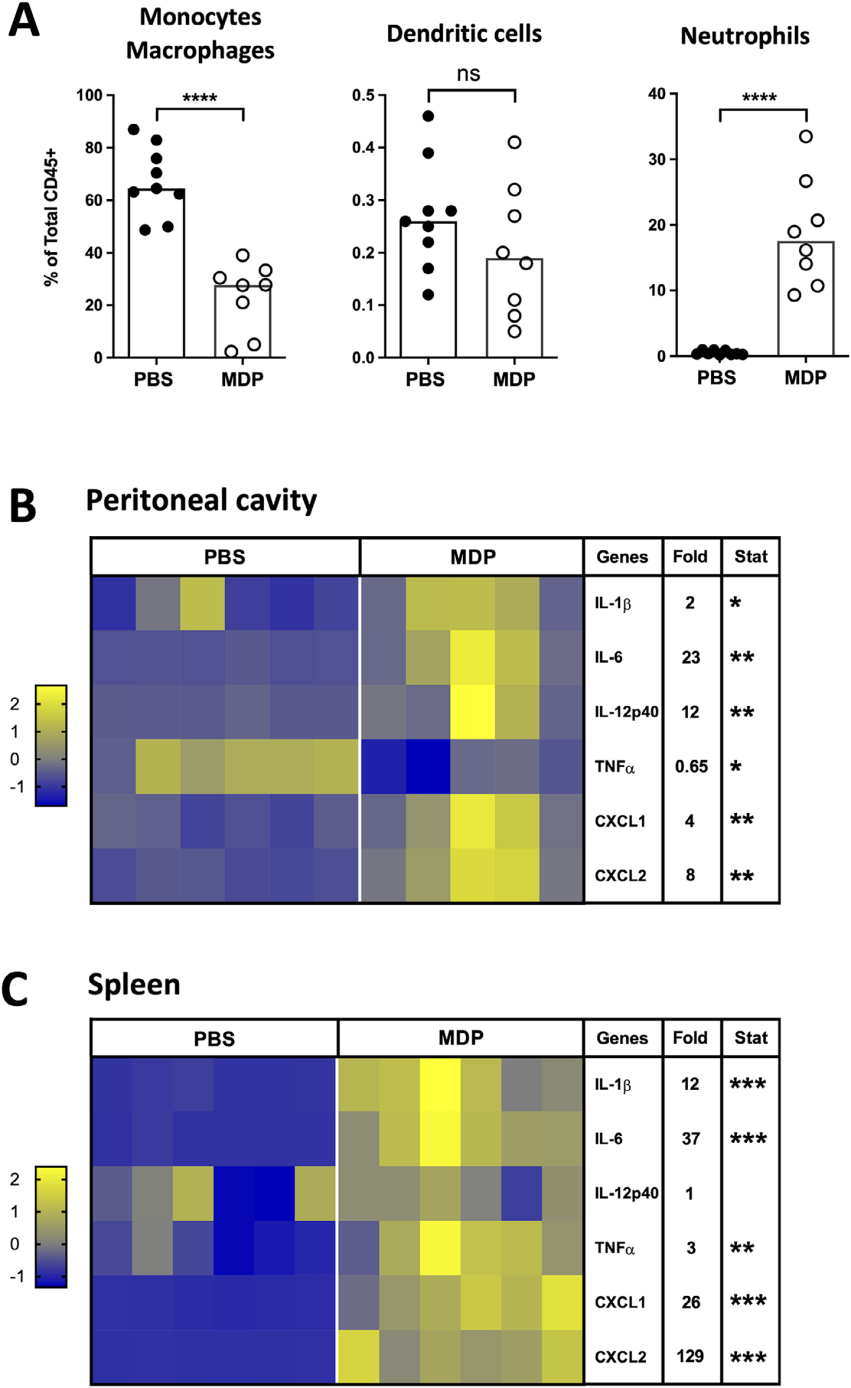
MDP injection into *Cryptosporidium parvum*–infected‐neonatal mice increases the inflammatory response in the peritoneal cavity and in the spleen. PND3 neonatal mice were orally infected with 5.10^5^ oocysts of *C. parvum* and received 200 µg of MDP by IP route at Day 5 p.i. After 4 h, cells from the peritoneal cavity were collected and analyzed by flow cytometry (A) and by qRT‐PCR (B). The percentage of monocytes/macrophages CD45+ CD11c+ MHCII+ CD64+, dendritic cells CD45+ CD11c+ MHCII+ CD64−, and neutrophils CD45+ Ly6G+ CD11b+ was determined (*n* = 8–9 mice for each group). The data are representative of two independent experiments. Each point corresponds to one mouse, and bars represent the median of each group. After 4 h, spleens were sampled and analyzed by q‐RT‐PCR (C). (B and C) Levels of pro‐inflammatory gene expression were quantified by RT‐qPCR. Heat maps are designed by *z*‐score of the 2^−Δ^ *^Ct^* results for each gene, and fold change of mRNA expression calculated as 2^−Δ^ *^Ct^* results of the MDP‐group in comparison to the PBS‐group (*n* = 5–6 mice for each group). The data are representative of 2–3 independent experiments. Statistics are calculated by the Mann–Whitney test. MDP, muramyl dipeptide; PND, postnatal days. ns > 0.05, *p < 0.05, **p < 0.01, ***p < 0.001, ****p < 0.0001.

**FIGURE 3:**
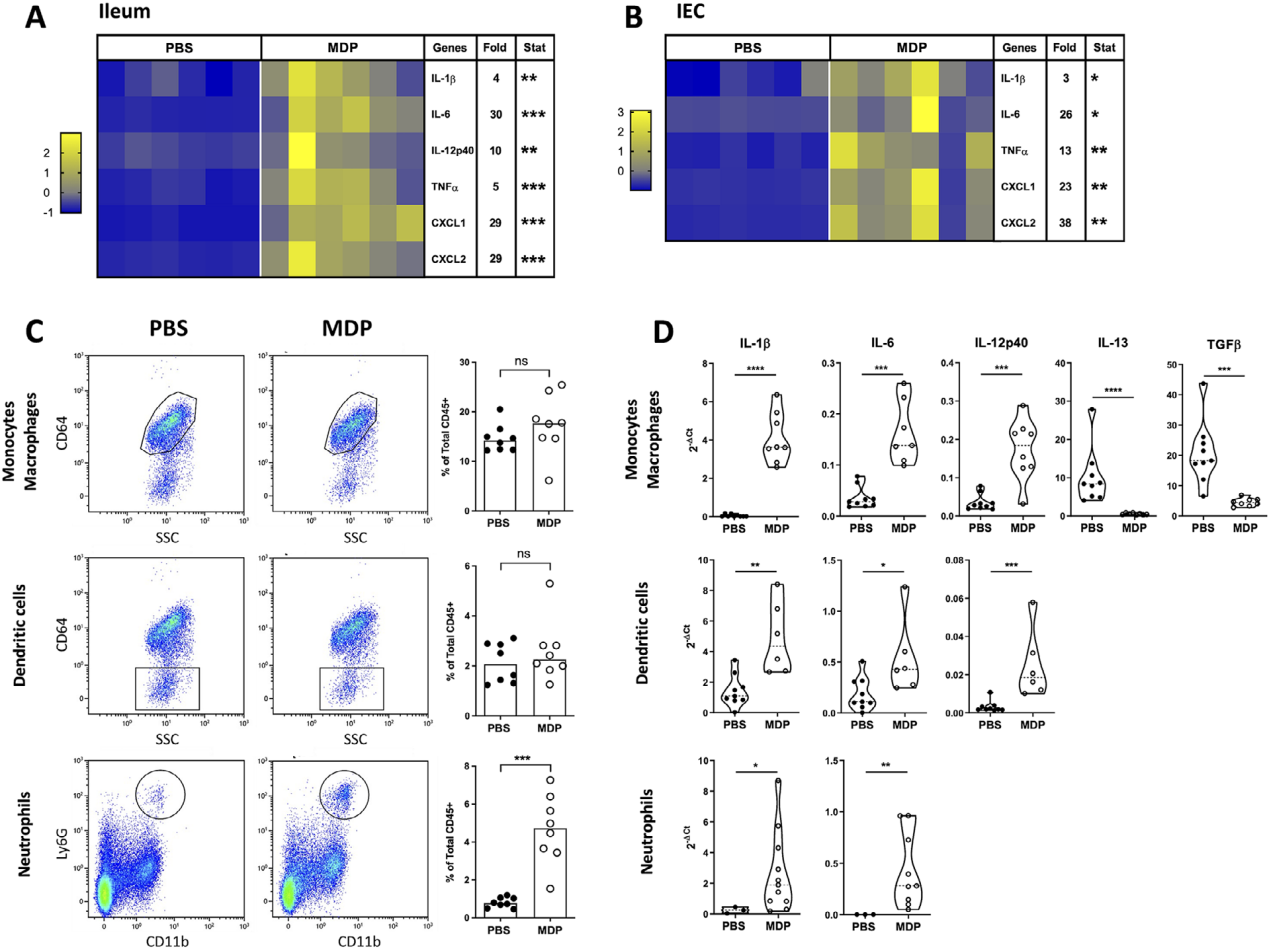
The systemic injection of NOD2 agonist increases inflammation in the ileum of *Cryptosporidium parvum*–infected‐neonatal mice. PND3 neonatal mice were orally infected with 5 × 10^5^ oocysts of *C. parvum* and received 200 µg of MDP by IP route at Day 5 p.i. Four hours after MDP injection, the intestine was sampled. The levels of inflammatory gene expression were quantified by RT‐qPCR in the ileum tissue (A) and in purified IECs (B). Heat maps are designed by *z*‐score of the 2^−Δ^ *^Ct^* results for each gene, and fold change of mRNA expression calculated as 2^−Δ^ *^Ct^* results of the MDP‐group in comparison to the PBS‐group (*n* = 6 mice for each group). (C) Cells from the *lamina propria* of the distal small intestine were collected and analyzed by flow cytometry. The percentage of monocytes/macrophages CD45+ CD11c+ MHCII+ CD64+, dendritic cells CD45+ CD11c+ MHCII+ CD64−, and neutrophils CD45+ Ly6G+ CD11b+ was determined (*n* = 8 mice for each group). The data for A–B–C are representative of three independent experiments. (D) Monocytes/macrophages, dendritic cells, and neutrophils were sorted, and the levels of inflammatory gene expression were quantified by RT‐qPCR. Results are expressed as the 2^−Δ^ *^Ct^* results for each gene and compared between the MDP‐group and the PBS‐group (*n* = 9–11 mice for each group). The data are representative of one independent experiments. Statistics are calculated by the Mann–Whitney test. IEC, intestinal epithelial cell; MDP, muramyl dipeptide; PND, postnatal days. ns > 0.05, *p < 0.05, **p < 0.01, ***p < 0.001, ****p < 0.0001.

We have previously shown that a transient increase in the inflammatory response induced by TLR stimulation can help to reduce *C. parvum* infection in neonatal mice [11, 12]. Here, upon MDP injection by IP route into C. parvum‐infected neonatal mice, a significant increase in the percentage of neutrophils (CD45+ CD11b+ Ly6G+) was observed in the peritoneal cavity 4 h after injection. In contrast, the percentage of macrophages (CD45+ CD11c+ MHCII+ CD64+) significantly decreased, whereas the percentage of DC (CD45+ CD11c+ MHCII+ CD64−) remained unchanged (Figure 2A). This shift of immune cell percentage was associated with a significant increase in the gene expression of some pro‐inflammatory cytokines and chemokines (*IL‐1β*, *IL‐6, IL‐12p40, CXCL1*, and *CXCL2*) in total of intraperitoneal cells (Figure 2B), with the exception of *TNF‐α* expression being decreased. This increased inflammatory response was also observed at the systemic level within the spleen, exhibiting a substantial increase in *IL‐1β*, *IL‐6*, *TNF‐α, CXCL1*, and *CXCL2* (Figure 2C). In the ileum tissue, at the site of infection of C. parvum, similar to the peritoneal cavity and the spleen, MDP injection significantly increased the gene expression of *IL‐1β*, *IL‐6, TNF‐α, IL12‐p40, CXCL1*, and *CXCL2* (Figure 3A), and also in purified IECs (Figure 3B). Twenty‐four hours after MDP injection, these modifications of gene expression were no more visible (Figure S4A,B).

The inflammatory state of the intestine after MDP injection was evident by neutrophils recruitment as early as 4 h and was still visible 24 h after injection (Figure 3C and Figure S4C). In contrast, no changes were observed in the percentage of monocytes/macrophages (CD45+ CD11c+ MHCII+ CD64+) and of dendritic cells (CD45+ CD11c+ MHCII+ CD64−) (Figure 3C). Sorting neutrophils, monocytes/macrophages, and dendritic cells from the ileum of infected neonatal mice revealed increased mRNA gene expression of *IL‐1β* and *IL‐6* in each cell population after MDP injection (Figure 3D). Increased *IL‐12p40* mRNA gene expression was observed in dendritic cells and monocytes/macrophages from MDP‐injected neonatal mice. In addition, the monocytes/macrophages expressed less *IL‐13* and *TGFβ* mRNAs (Figure 3D). These results may indicate a shift in resident macrophages toward a pro‐inflammatory profile, characteristic of “M1” macrophages. Altogether, these data demonstrate that MDP injection into neonatal mice stimulates a rapid and transient inflammatory response both at the injection and infection sites as well as in the spleen.

### 2.3 Impact of MDP Injection on IECs in Neonatal Mice

**FIGURE 4:**
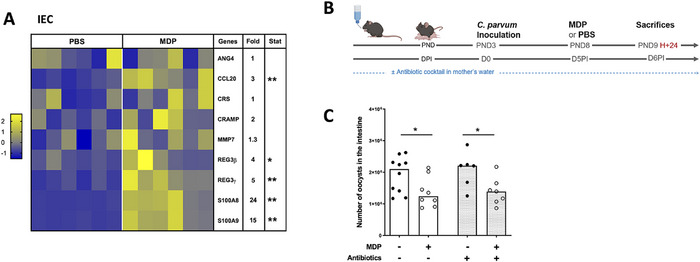
Microbiota is not involved in the MDP‐induced protection despite a modified response of antimicrobial response in neonatal mice. PND3 neonatal mice were orally infected with 5 × 10^5^ oocysts of *Cryptosporidium parvum* and received 200 µg of MDP by IP route at 5 d.p.i. Mice were sacrificed 4 h postinjection to evaluate the response in antimicrobial peptides of the purified IECs by RT‐qPCR (A). Heat map is designed by *z*‐score of the 2^−Δ^ *^Ct^* results for each gene and fold change of mRNA expression calculated as 2^−Δ^ *^Ct^* results of the MDP‐group in comparison to the PBS‐group (*n* = 6 mice for each group). The data are representative of three independent experiments. (B) Experimental timeline. Pregnant mice received a mixture of antibiotics in their drinking water 2 days before the expected day of delivery and throughout the experiment to reduce their gut intestinal bacterial burden and that of their pups. (C) Parasite load in the intestine was evaluated 24 h later (*n* = 7–10 mice for each group). The data are representative of one experiment. Each point corresponds to one mouse and bars represent the median. Statistics are calculated by the Mann–Whitney test. DPI, days post‐inoculation; IEC, intestinal epithelial cell; MDP, muramyl dipeptide; PND, postnatal days. ns > 0.05, *p < 0.05, **p < 0.01.

NOD2 receptor is expressed by various immune cells and IECs [20], and its stimulation is known to trigger the secretion of the antimicrobial peptides. Significant overexpression of the antimicrobial peptides *Reg3β/γ* and *S100A8/A9* genes was observed in the IECs of MDP‐injected neonatal mice compared to PBS‐injected neonatal mice (Figure 4A). These data suggest that IECs may directly respond to MDP, as described previously with intestinal organoid models [32], or to the inflammatory environment induced by MDP, thus contributing to protection. Considering the modification in antimicrobial peptide responses after MDP injection, one may hypothesize that microbiota could play a role in reducing the intestinal parasite load as previously described after PolyI:C injection [12]. To investigate this, we used an experimental model in which pregnant mice received a mixture of broad‐spectrum antibiotics in their drinking water 2 days before the expected day of delivery and throughout the experiment in order to reduce the bacterial loads in their intestines and that of their pups (Figure 4B). However, upon the administration of MDP to neonates born from antibiotic‐treated dams, the reduction in the parasite load was not significantly modified compared to conventional neonates, suggesting that the microbiota is not involved in the protection induced by MDP (Figure 4C).

**FIGURE 5:**
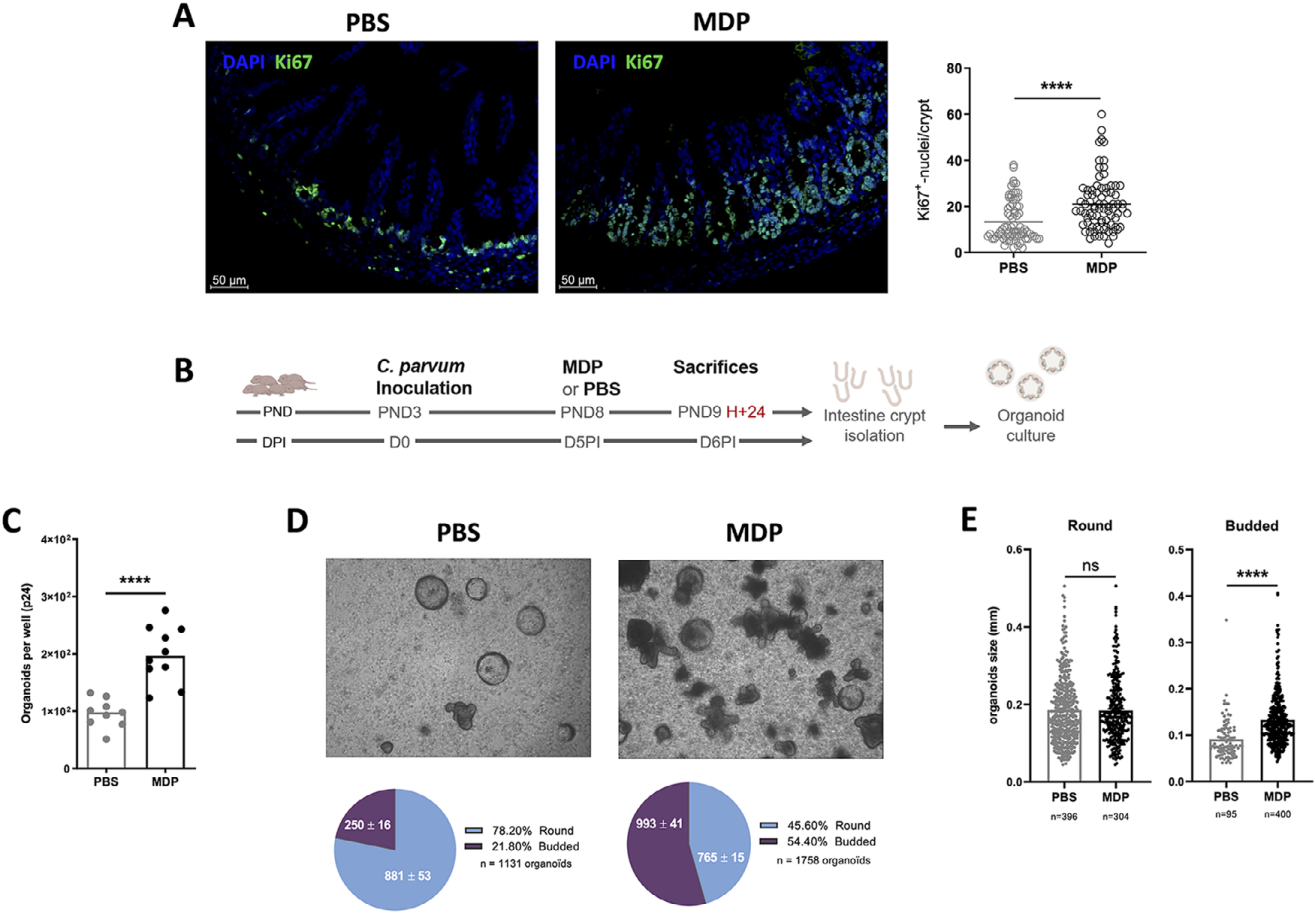
MDP injection promotes intestinal epithelial regeneration in neonatal mice infected by *Cryptosporidium parvum*. PND3 neonatal mice were orally infected with 5 × 10^5^ oocysts of *C. parvum* and received 200 µg of MDP by IP route at 5 d.p.i. Mice were scarified 24 h postinjection to evaluate the impact on IECs. (A) The staining of Ki67+ cells in the ileum of infected mice with or without MDP treatment was quantified by the number of Ki67+ cells per crypt in three separate areas for each animal (*n* = 3 animals/condition) (right panel). In the left panel, immunofluorescence staining of neonatal mice 40×, with Ki67+ cells (green) and nuclei (DAPI, blue). (B) Experimental timeline to generate organoids. Intestinal crypts were isolated from MDP‐ or PBS‐infected mice (*n* = 5 per group) to generate and culture intestinal organoids for 6 days (2 wells of a 24‐well per animal). The numbers (C), the state of development (D), and the size of organoids (E) were determined. The data derive from two independent experiments. Statistics are calculated by the Mann–Whitney test. DPI, days post‐inoculation; MDP, muramyl dipeptide; PND, postnatal days. ns > 0.05, ****p < 0.0001.

Among IECs, Lgr5+ stem cells constitutively express NOD2 receptor at a higher level compared to Paneth cells and other epithelial cells, contributing to their protection and epithelial regeneration [20, 23, 33]. In neonatal mice infected with C. parvum that received MDP, a significant hyperproliferation of intestinal stem cells was observed after Ki67+ staining (Figure 5A). We next isolated small intestinal crypts from neonatal mice injected with PBS or MDP and cultured them in Matrigel to allow intestinal organoid development (Figure 5B). The organoids were allowed to mature in vitro for 6 days before being counted and characterized. Following in vivo stimulation of mice with MDP, a significant increase in the number of organoids was observed (Figure 5C), and there was an increased proportion of budded organoids, indicating enhanced maturation and regeneration of the epithelium (Figure 5D). Furthermore, the size of budded organoids increased when derived from intestinal crypts isolated from MDP‐injected neonatal mice (Figure 5E). Taken together, these data clearly demonstrate that injection of MDP into C. parvum‐infected neonatal mice stimulated, either directly or indirectly, stem cell proliferation and promoted epithelium regeneration, which could explain the elimination of infected cells, and therefore the reduction in parasite numbers.

### 2.4 IFN‐γ Is a Key Cytokine Involved in the Mechanism Underlying the Protection Induced by MDP in Neonatal Mice

**FIGURE 6:**
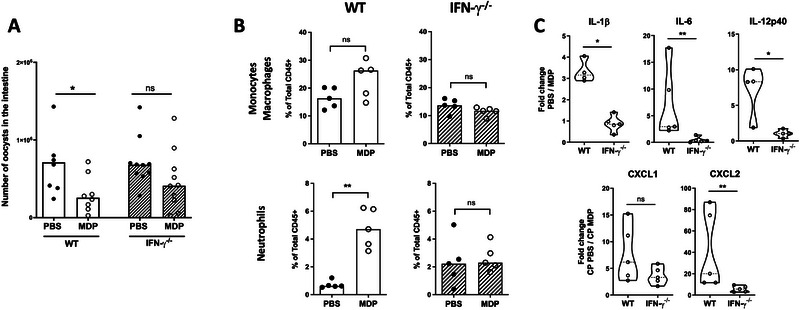
The reduced *Cryptosporidium parvum* intestinal load induced by MDP into neonatal mice is dependent on IFN‐γ. PND3 WT or IFN‐γ^−/−^ neonatal mice were orally infected with 5 × 10^5^ oocysts of *C. parvum* and received 200 µg of MDP by IP route at 5 d.p.i. (A) The intestinal parasite load in the neonatal mice was evaluated 24 h after MDP injection (*n* = 7–10 mice for each group). (B and C) Four hours after MDP‐injection, intestines were collected and analyzed by flow cytometry and by qRT‐PCR. (B) Cells from the intestinal *lamina propria* were collected and analyzed by flow cytometry. The percentage of monocytes/macrophages (CD45+ CD11c+ MHCII+ CD64+) and neutrophils (CD45+ Ly6G+ CD11b+) was determined (*n* = 5 mice for each group). (C) Levels of inflammatory gene expression were quantified by RT‐qPCR in the ileum. Data are expressed by the fold change of mRNA expression calculated as 2^−Δ^ *^Ct^* results of MDP‐group in comparison to the PBS‐group of each mouse strain (WT or IFN‐γ^−/−^) (*n* = 4–5 mice for each group). The data are representative of two independent experiments. Each point corresponds to one mouse, and bars represent the median. Statistics are calculated by the Mann–Whitney test. MDP, muramyl dipeptide; PND, postnatal days. ns > 0.05, *p < 0.05,**p < 0.01.

IFN‐γ is a key cytokine essential for host resistance to *C. parvum* [34, 35]. Furthermore, it actively contributes to the reduction of parasite load in the intestine of neonatal mice upon stimulation with TLR ligands [11, 12]. Therefore, we injected MDP into IFN‐γ deficient neonatal mice and observed no significant reduction in parasite load compared to PBS‐IFN‐γ deficient neonatal mice, in contrast to what was observed in WT neonates (Figure 6A). As previously described, the number of neutrophils was higher in IFN‐γ‐deficient neonatal mice infected with *C. parvum* compared to WT neonatal mice [36]. However, following MDP injection in the absence of IFN‐γ, no neutrophil recruitment was observed in the ileum of neonatal mice, unlike WT mice (Figure 6B vs. Figure 3C). There was also no modulation of the percentage of monocytes/macrophages (Figure 6B), which is similar to the response in WT neonatal mice (Figure 3C).

This observation suggests a correlation between the presence of IFN‐γ and the recruitment of neutrophils. When the cytokine response was analyzed under these conditions, the fold increase in gene expression of *IL‐1β*, *IL‐6, IL‐12p40*, and *CXCL2* was significantly reduced in IFN‐γ deficient mice compared to wild type (Figure 6C). This suggests that the intestinal inflammatory environment induced by MDP contributes to the reduction of *C. parvum* load.

### 2.5 The IFN‐γ‐Dependent Production of the Cytokine IL‐22 Contributes to the Reduction of the Intestinal Parasite Load Induced by MDP Injection

**FIGURE 7:**
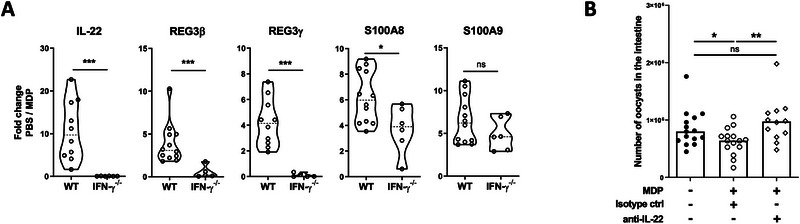
IL‐22 plays a role in the protection induced by MDP in *Cryptosporidium parvum*‐infected neonatal mice. (A) PND3 WT or IFN‐γ^−/−^ neonatal mice were orally infected with 5 × 10^5^ oocysts of *C. parvum* and received 200 µg of MDP by IP route at 5 d.p.i. Mice were sacrificed 4 h postinjection to evaluate the intestinal (ileal tissue) gene expression levels of *IL‐22, REG3β/γ*, and *S100A8/A9* by RT‐qPCR. Data are expressed by the fold change of mRNA expression calculated as 2^−Δ^ *^Ct^* results of MDP‐group in comparison to the PBS‐group of each mouse strain (WT or IFN‐γ^−/−^) (*n* = 4–5 mice for each group). (B) PND3 WT neonatal mice were orally infected with 5 × 10^5^ oocysts of *C. parvum*, and mice received 200 µg of MDP and an anti‐IL‐22‐neutralizing antibody or isotype control antibody by IP route at 5 d.p.i. Mice were sacrificed 24 h postinjection to evaluate the parasite load in the intestine (*n* = 12–15 mice for each group). The data are representative of one independent experiments. Each point corresponds to one mouse, and bars represent the median. Statistics are calculated by the one‐way Kruskal–Wallis test followed up by Dunn's correction. MDP, muramyl dipeptide; PND, postnatal days. ns > 0.05, *p < 0.05,**p < 0.01, ***p < 0.001.

The increased response of *Reg‐3β/γ* and *S100A8/A9* in IECs and the observed epithelial regeneration in the small intestine of neonatal mice following MDP injection (Figure 4A) prompted us to investigate the role of IL‐22 in this process. In fact, it is well documented that IL‐22 induces the production of innate antimicrobial molecules, including defensins, Reg family molecules and S100 proteins [37, 38, 39, 40] stimulate the wound healing of intestinal tissue [41, 42, 43, 44]. We first examined the mRNA response of IL‐22 in ileal tissue and observed an approximately 10‐fold increase after MDP injection (Figure 7A). This intestinal increased of *IL‐22* mRNA gene expression post‐MDP injection was not observed in the absence of IFN‐γ (Figure 7A) suggesting its involvement in the IFN‐γ‐dependent protective mechanism induced by MDP. Consistently, the increased *Reg‐3β/γ* and *S100A8* mRNA expression was also diminished in IFN‐γ deficient mice after MDP injection compared to WT neonatal mice (Figure 7A). As IECs are the only cells in the intestine that express the IL‐22 receptor [37, 45], and that also express antimicrobial peptides after MDP injection in infected neonatal mice, as shown in Figure 4A, we further investigated a possible direct protective effect of IL‐22 on infected epithelial cells as has been described for other cytokines, such as IFN‐γ, TNF‐α, and type I IFN [46, 47, 48]. We then analyzed the role of IL‐22 in vivo by injecting a neutralizing antibody against IL‐22 and observed a loss of protection induced by MDP injection (Figure 7B).

Altogether, these data demonstrate that IFN‐γ contributes to an immune loop involving neutrophils, IL‐22, Reg3β/γ, and S100A8/A9 that helps to reduce *C. parvum* intestinal load after MDP injection.

## 3 Discussion

Early life is characterized by increased susceptibility to respiratory and enteric infections. In humans, neonatal infections remain a major concern, with 5 million children under the age of 5 succumbing from infectious diseases in 2021 (United Nations Inter‐Agency Group for Child Mortality Estimation [UN IGME] 2023) [49]. Similarly, respiratory and intestinal infections in calves and piglets result in significant economic losses in livestock production. High morbidity rates have been reported, such as diarrhea (calves ≤ 35%; piglets ≤ 50%) and respiratory diseases (calves ≤  80%; piglets ≤ 40%) [50]. This vulnerable period is characterized by quantitative and qualitative defects in immunity, particularly at mucosal surfaces, where environmental, nutritional, and microbial antigens shape the immune system. Newborns, lacking a fully developed adaptive immune system, rely on innate immune responses and maternally transmitted immunity for early protection against pathogens [51]. Enhancing protection against infection and disease by modulating the innate immune response is therefore a feasible approach.

Modulation of TLRs and associated pathways presents opportunities to improve pathogen recognition and modulate the inflammatory response to combat infection. As an example, prophylactic TLR2 activation can prime airway immunity for enhanced protection against rhinovirus, influenza, and SARS‐CoV‐2 infection [52, 53]. In addition, PolyI:C treatment to stimulate TLR3 has been described to effectively promote the clearance of hepatitis B virus (HBV) infection [54]. In neonates, the benefits of administering TLR agonists (e.g., resiquimod and LPS) have been demonstrated in a neonatal mouse model predisposed to poor sepsis outcomes [55]. In addition, we have previously demonstrated the benefits of TLR9 and TLR3 stimulation by administration of CpG‐ODN and PolyI:C, respectively, to induce a strong intestinal innate immune response and efficiently control cryptosporidiosis in the neonatal mouse model [11, 12].

In this study, we have demonstrated that MDP, the agonist of the NOD2 receptor in neonatal mice can stimulate both systemic and intestinal inflammatory responses, resulting in a rapid renewal of the intestinal epithelium, which transiently helps to clear *C. parvum* from the intestine. Mechanistically, mice were protected upon MDP injection in an IFN‐γ and IL22‐dependent manner. Similarly, NOD2 activation by bacterial MDP has previously been described to suppress viral infection by human cytomegalovirus (HCMV) via an IFN‐β‐dependent pathway [56] and to protect from oxazolone‐induced colitis by stimulating hematopoietic cells [57]. Our previous studies, using TLR3 and TLR9 ligands to stimulate the innate immune system of neonatal mice and protect them against cryptosporidiosis, highlighted the crucial role of MyD88 signaling activation in dendritic cells. When TLR3‐TRIF signaling pathway is activated by PolyI:C, the presence of microbiota is required to allow the simultaneous MyD88‐activation via a TLR5 recognition [12]. We therefore hypothesized that microbiota may also contribute to the decrease of infection induced by MDP because the presence of intestinal microbiota also enables the more or less efficient absorption of bacterial PGNs, such as MDP [58]. In this study, the antibiotic‐induced microbiota modifications had no effect in the MDP‐induced protection whatever the immune defects described in neonates when mothers received antibiotics during pregnancy [59]. These results suggest that activation of the NOD2 signaling pathway is sufficient to promote parasite clearance.

*Cryptosporidium* affects epithelial turnover by promoting the proliferation and migration of epithelial cells, resulting in an increased expulsion of cells from the epithelial layer. Furthermore, increased epithelial proliferation correlates with the parasite burden in mice [60]. As Wallbank et al. suggest, this enhanced extrusion is likely a means of eliminating infected cells even if the parasite is resistant to cell death. Our data demonstrate increased proliferation in the cell crypt of infected neonates who received MDP compared to infected neonates, supporting this hypothesis. In the intestine, NOD2 receptor is expressed in immune and epithelial cells, and more strongly in Paneth cells and stem cells [20]. This suggests that MDP may directly impact the intestinal epithelium, favoring the elimination of the infection. However, other parasite elimination mechanisms induced by MDP cannot be excluded, such as the impact of IFN‐γ, which can directly inhibit *C. parvum* development after infection [48, 61] or the induction of autophagy [62, 63]. Furthermore, cytokines, such as IL‐6, IL‐22, and IL‐17, can contribute to intestinal epithelial proliferation [64]. By culturing intestinal crypts isolated from infected neonatal mice that had or had not received MDP, we confirmed in vitro the effect of MDP injection on the proliferation of IECs. In the absence of immune cells in the organoid cultures, and as the culture was initiated with the same number of intestinal crypts, the stem cell niche environment may also contribute to the proliferation of intestinal crypt cells.

In the present study, the reduced infection of *C. parvum* induced in neonatal mice by MDP injection was dependent on IFN‐γ, and its deficiency was associated with a diminished recruitment of neutrophils and an altered response in IL‐22 and Reg3γ. To allow MDP to be delivered to the intracellular receptor NOD2, the di–tripeptide transporter PepT1 plays a critical role [65, 66]. Interestingly, the expression of the transporter [67] remains stable even during intestinal mucosal injury [68, 69, 70, 71]. Its expression can be upregulated by IFN‐γ [72], but also in the context of *C. parvum* infection in a rat model [73]. Taken together, these data may provide new insights in the IFN‐γ‐dependent mechanism of protection induced by MDP administration, as described in this study.

We demonstrated that NOD2 stimulation by MDP in neonates is associated with a rapid influx of neutrophils into the intestine, alongside with an increased response of CXCL1 and CXCL2 by IECs, whereas the proportion of macrophages remains unchanged. Neutrophils are recruited into the intestine after *C. parvum* infection [7, 74, 75], but their role in the control of cryptosporidiosis is still poorly understood. In an in vitro study, neutrophils were shown to form NETs that resulted in the entrapment of 15% of sporozoites [76]. However, in vivo depletion of neutrophils in the piglet did not affect the severity or pathology of infection [75]. In the SCIDbgMN mouse model, which is characterized by a depletion of functional macrophages and neutrophils in an SCIDbg background already lacking T, B, and NK cells, Takeuchi et al. reported a more severe parasite load in the early phase of *C. parvum* infection, leading to mouse mortality [77]. Neutrophils have been proposed to convert resident “M2” macrophages to an “M1” macrophage phenotype, contributing to the mounting of a protective immune response [77]. Their role in cryptosporidiosis may therefore be less direct and more in cooperation with other cell populations. In the present study, when neutrophils and macrophages were sorted to analyze their transcriptional profile, both cell types exhibited higher levels of *IL‐1β* and *IL‐6* after MDP injection, and macrophages decreased their *TGF‐β* and *IL‐13* mRNA response. These data may be related to a potential conversion of macrophages toward a pro‐inflammatory profile associated with the neutrophil influx in the intestine of neonates after NOD2 stimulation, which may help to reduce *C. parvum* infection.

We also reported a robust IFN‐γ‐dependent production of IL‐22 after MDP injection in the intestine of neonates, which contributed to the reduction of the parasite burden. Surprisingly, IL‐22 expression in neutrophils of neonatal mice was not significantly modified by MDP injection (Figure S5). However, consistent with the lack of neutrophil recruitment in IFN‐γ deficient mice after MDP injection, it could be suggested that the increased intestinal IL‐22 response is due to the increased number of neutrophils. Several studies have described that IL‐22 induces the production of antimicrobial peptides such as Reg3γ and is involved in the recovery of the intestinal epithelial barrier after acute injury by promoting the proliferation of LGR5+ intestinal epithelial stem cells. In addition, IL‐22‐mediated regulation of epithelial function closely parallels that of other pro‐inflammatory cytokines, such as IFN‐γ and TNF‐α [78]. We observed a significant increase in the Reg3γ mRNA after MDP injection, which was reduced in the absence of IFN‐γ, where the IL‐22 response was also significantly reduced. In addition, we described that the systemic injection of MDP in neonates affected intestinal epithelial stem cell proliferation. Taken together, these data suggest that maintaining intestinal homeostasis through IL‐22 in an IFN‐γ‐dependent manner may promote the removal of infected cells by renewing of the intestinal epithelium, as suggested by the IL‐22/Reg3γ loop.

To the best of our knowledge, we have demonstrated for the first time that stimulating the NOD2 receptor with MDP can help to clear a *C. parvum* intestinal infection in neonatal mice. Future studies are essential in order to investigate the precise role of IL‐22 in protecting against cryptosporidiosis and maintaining an efficient immune response.

## 4 Materials and Methods

### 4.1 Ethic Statements

All experimental protocols were performed in accordance with French legislation (Décret: 2001‐464 29/05/01) and EEC regulations (86/609/CEE) on the care and use of laboratory animals, after validation by the local ethics committee for animal experimentation Comité d'Ethique pour l'Expérimentation Animale Val de Loire (CEEA VdL no. 019: APAFIS#34587, APAFIS#21515). The mice were housed in a specific pathogen‐free animal facility in the Infectiology of Farm, Model and Wildlife Animals Facility (PFIE, Centre INRAE Val de Loire: https://www6.val‐de‐loire.inrae.fr/pfie/ member of the National Infrastructure EMERG'IN). They were housed in individual ventilated cages with HEPA filters, with food and water ad libitum. All personnel involved in animal work received specific training in animal care, handling, and experimentation, as required by the French Ministry of Agriculture.

### 4.2 Parasite Preparation

Oocysts of *C. parvum* were originally obtained from an infected child (INRAE *C. parvum*). This strain was continuously maintained by regular passages in newborn calves at the PFIE Centre INRAE Val de Loire facility, and oocysts were purified following a protocol previously described [34]. Transgenic INRAE *C. parvum* expressing nano‐luciferase [79] was used, for in vitro experiments. This strain was maintained by regular passages in IFN‐γ^−/−^ mice.

### 4.3 Mouse Infection and Experimental Models

Specific‐pathogen‐free C57BL/6J mice (WT and interferon‐gamma knockout (IFN‐γ^−/−^)) were used in this study. Three‐day‐old neonatal mice (PND3 for postnatal day) were orally inoculated with 5 × 10^5^ oocysts of *C. parvum*. Five days postinoculation (d.p.i.), at the end of the first cycle of parasite multiplication and just before the peak of infection (8–10 d.p.i.), neonatal mice received NOD ligands or PBS, and parasitic load was determined 24 h later. The level of infection in individual neonatal mice was assessed by counting the number of oocysts in the whole intestine, as previously described [36]. NOD1 agonist, iEDAP: 200 µg (tlrl‐dap, InvivoGen), NOD2 agonist, MDP: 5, 50, and 200 µg (tlrl‐mdp, InvivoGen), MDP control: 200 µg (MDP Ct) (tlrl‐mdpcl, InvivoGen) were used in this study. To decipher the innate immune response, infected mice were sacrificed 4 or 24 h after NOD ligand administration, and different samples were collected for further analysis: ileum, spleen, and peritoneal cells.

To study the impact of microbiota in the MDP‐induced‐protection mechanism, dams of newborns were treated with an antibiotic solution dissolved in drinking water from 2 days before delivery until analysis of the neonates at 6 d.p.i. The antibiotic solution was composed of ampicillin (1 mg/mL), vancomycin (0.5 mg/mL), colistin (1 mg/mL), streptomycin (5 mg/mL) (Antibiotics, Sigma‐Aldrich), and 2.5% (wt/vol) sucrose. To evaluate the role of IL‐22, infected mice received simultaneous intraperitoneal injections of 200 µg of MDP and 10 µg of mouse IL‐22 antibody (R&D Systems) or an equivalent amount of isotype IgG from goat (R&D Systems), whereas control mice received PBS.

### 4.4 Immune Cell Preparation

Peritoneal cells were obtained from the peritoneal lavage of PND8 C57BL/6 mice. Peritoneal cells were collected by injecting 4 × 500 µL of RPMI into the peritoneal cavity. The cells were then passed through a 100‐µm strainer and centrifuged. Finally, the cells were distributed in round‐bottom 96‐well plates (Falcon) for flow cytometry analyses or were frozen at −80°C with TRI Reagent (Sigma‐Aldrich) for future transcriptomic studies. To isolate immune cells from *lamina propria*, jejunum, and ileum were cut longitudinally, washed with RPMI medium, placed in ice‐cold Cell Recovery Solution (Corning), and incubated for 3 h at 4°C. The intestines were then vigorously shaken to separate the IECs from the *lamina propria*. The contents were filtered through a 100‐µm cell strainer to isolate the IECs. The IECs were centrifuged at 400 *g* for 10 min at 4°C, and the pellets were frozen at −80°C with TRI Reagent (Sigma‐Aldrich) and preserved for future transcriptomic studies. The intestines retained on the filters were mechanically cut into 0.5 cm pieces and enzymatically digested in RPMI 10% FCS with 100 U/mL of collagenase type I (Sigma‐Aldrich) for 1 h at 37°C. After digestion, cells were filtered through 200 µm filters and centrifuged at 400 *g* for 10 min at 4°C. Pellets were washed with 10% FCS in PBS, and cells were collected with PBS 2% FCS 1% mouse serum to block nonspecific staining. Finally, *lamina propria* cells were distributed in round‐bottom 96‐well plate (Falcon) for flow cytometry analysis or cell sorting.

### 4.5 Flow Cytometry and Cell Sorting

Isolated cells were stained with a mix of antibodies in an FACS medium (PBS, 2% FCS, 2 mM EDTA) for 30 min in the dark at 4°C. The mix contained anti‐mouse CD45‐BV711 (BioLegend); MHCII‐BV421 (BioLegend); CD11c‐FITC (BioLegend); CD64‐PE (Miltenyi Biotec); CD11b‐APC (BioLegend); and Ly6G‐PeCy7 (BioLegend). Cells were then washed twice, then fixed with fixation buffer (BD Biosciences) for flow cytometry, or suspended in PBS, 2% FCS, 2 mM EDTA for cell sorting. Cells were analyzed on an LSR Fortessa X‐20 (BD Biosciences) or sorted using an high‐speed cell sorter MoFlo Astrios EQ (Beckman Coulter). Flow cytometry data were analyzed using Kaluza software (Beckman Coulter). Sorted cells were stored for future transcriptomic studies. Figure S2 shows the gating strategy used for the analyses.

### 4.6 RNA Extraction and qRT‐PCR

At sacrifice, spleen and ileum tissues were frozen in nitrogen liquid and stored at −80°C. Total RNAs (spleen, ileum, and peritoneal cells) were extracted using Trizol reagent solution (Sigma‐Aldrich) and processed according to the manufacturer's recommendations. Reverse transcriptase (RT) reaction was performed using oligo(dT)18 primers and random hexamers with M‐MLV reverse transcriptase (Promega). RT‐qPCR reaction consisted of 7.5 µL of SYBR Green (BIO‐RAD), 0.5 µM of forward primer, 0.5 µM of reverse primer, and RNase DNase‐free water up to 13 µL. Finally, 20 ng of cDNA samples were added. Quantitative RT‐PCR was performed on a CFX96 instrument (Biorad), and the cycling steps were 5 min at 95°C, followed by 40 cycles of 10 s at 95°C, 15 s at 60°C, and a melting curve phase. Expression levels were calculated using the formula 2^−Δ^ *^Ct^*, where Δ*Ct*  = * Ct* gene − *Ct* average housekeeping for the control sample. HPRT and PPIA were used as internal standards for normalization. Primers for gene quantification are described in Figure S3. Heat maps are constructed using the *z*‐score of the 2^−Δ^ *^Ct^*.

### 4.7 Immunofluorescence Imaging and Analysis

Samples of ileum tissues were fixed overnight at 4°C in a fresh solution of 4% paraformaldehyde (Sigma‐Aldrich) in PBS. Samples were then washed for 1 day in PBS and incubated in a solution of 30% sucrose‐PBS (Sigma‐Aldrich) for at least 3 h. Ilea were included in paraffin blocks and sliced. Sections were de‐waxed by an automate Leica ST5020 Multistainer (Leica biosystems) and incubated in a sodium citrate buffer (pH = 6.12) for 20 min for antigen retrieval. Sections were next permeabilized, and Fc‐receptors were blocked using a solution of PBS, 0.1% TritonX‐100, 10% BSA for 20 min at room temperature. Sections were stained for 1 h at room temperature using a primary antibody rabbit anti‐Ki67 (Abcam) at 1/100 in PBS, 0.1% Triton, 1% BSA. After washing, a secondary antibody, swine anti‐rabbit FITC (DAKO) was added at 1/100 in PBS, 0.1% Triton, 1% BSA for 1 h at room temperature. Cell nuclei were counterstained with DAPI 1/2000 for 10 min at room temperature. Slides were mounted using Fluoromount‐G medium (Invitrogen). The acquisitions were performed under a widefield fluorescence microscope (Zeiss Axiovert 200M, Zeiss). Acquisitions were realized using a 40× objective using Zen Imaging software (Zeiss). Images were taken from three separate areas for each animal and blindly analyzed with the ImageJ software (version 2.9.0/1.53t, https://imagej.nih.gov). A semiautomated homemade macro was used to quantify the number of Ki67^+^‐nuclei per crypt. Briefly, crypts were manually segmented. Crypts were individually cropped, and StarDist [80, 81] (version 0.8.3, https://github.com/stardist) was used to segment nuclei on the basis of DAPI signal. By applying a threshold to the Ki67 signal, the number of proliferative cells per crypt was determined and compared between conditions.

### 4.8 Isolation of Intestinal Crypts and Organoid Culture

Sections of ileal segments were removed from infected mice that received or not MDP and placed in PBS without Ca^2+^ and Mg^2+^ (Thermo Fisher Scientific) supplemented with 100 U/mL penicillin and 100 mg/mL streptomycin. Intestinal crypts were isolated according to established protocols described previously [82, 83]. The tissue was opened longitudinally and cut into pieces. These pieces were then immersed in a dissociation buffer consisting of PBS without Ca^2+^ and Mg^2+^ containing 9 mM EDTA (Sigma Aldrich), 3 mM 1,4‐Dithiothreitol (DTT, Sigma Aldrich) and 10 µM Y27632 (Tocris) to dissociate intestinal crypts during 45 min of continuous shaking at 16 rpm at room temperature. The intestinal fragments were then transferred to a cold PBS solution without Ca^2+^ and Mg^2+^ and gently shaken by hand in an up‐and‐down motion for 2 min. To eliminate the intestinal villi, the supernatant was first passed through a 100 µm cell strainer and then rinsed with PBS without Ca^2+^ and Mg^2+^. This process was repeated using a 70‐µm cell strainer, and the crypts were then centrifuged at 220 *g* for 5 min at room temperature. The pellet containing intestinal crypts was suspended in DMEM/F12‐Glutamax (Gibco Life Technologies), 1% HEPES (Gibco Life Technologies), 5% FBS, 100 U/mL penicillin, and 100 mg/mL streptomycin. Approximately 2500 crypts were seeded into 50 µL of 75% of Matrigel mixed with 25% complete medium (DMEM/F‐12 Glutamax (Gibco Life Technologies) supplemented with 50% L‐WRN CM (homemade as described before [83]), 10 mM HEPES (Gibco Life Technologies), B27 1× (Thermo Fisher Scientific), 50 ng/mL EGF (Sigma Aldrich), 500 nM A83‐01 (Tocris), 10 µM SB2022190 (Tocris), 10 nM gastrin I (Tocris), 1 mM *N*‐acetyl‐l‐cysteine (Sigma), and 10 µM Y27632 (Tocris)) and seeded into pre‐warmed 24‐well plates. After Matrigel polymerization, 500 µL of complete medium was added per well. The organoid medium was refreshed every 2–3 days with complete medium. Images were captured by inverted phase contrast microscopy (Olympus CK40) and with an OptikaB9 digital camera (Optika). The number, size, and development stage of the organoids were monitored using the Optikavision Lite 2.1 software.

### 4.9 Statistics

Statistical analyses were performed using the GraphPad Prism v8 software (GraphPad Software, San Diego, USA). The nonparametric Mann–Whitney *t*‐test was used to determine the significance between two groups. For comparisons involving more than two groups, the one‐way nonparametric test (Kruskal–Wallis) followed up by Dunn's correction test was used. *p* values less than 0.05 were considered statistically significant.

## Author Contributions

**Sonia Lacroix‐Lamandé**: conceptualization, data curation, formal analysis, funding acquisition, investigation, methodology, project administration, resources, supervision, validation, visualization, writing – original draft, writing – review and editing. **Mégane Fernandez**: data curation, investigation, methodology, resources, validation, visualization, writing – original draft, writing – review and editing. **Tiffany Pezier**: data curation, formal analysis, investigation, methodology, resources, validation. **Julien Pichon**: formal analysis, writing – review and editing. **Yves Le Vern**: resources, writing – original draft. **Catherine Werts**: funding acquisition, writing – review and editing.

## Conflicts of Interest

The authors declare no conflicts of interest.

## Supporting information




**Supporting File 1**: eji70080‐sup‐0001‐Figures.pdf

## Data Availability

The data that support the findings of this study are available from the corresponding author upon request.
