# Supplementary material for: Administration of the NOD2 Agonist MDP Reduces Cryptosporidium parvum Infection in Neonatal Mice Through IL‐22 Involvement
Source: Eur J Immunol. 2025 Oct 20;55(10):e70080. doi: 10.1002/eji.70080 (PMC12538031; doi:10.1002/eji.70080)
Supplement: Supplementary file 1 — Supporting File 1: eji70080‐sup‐0001‐Figures.pdf [file EJI-55-e70080-s001.pdf]

S1 Fig.

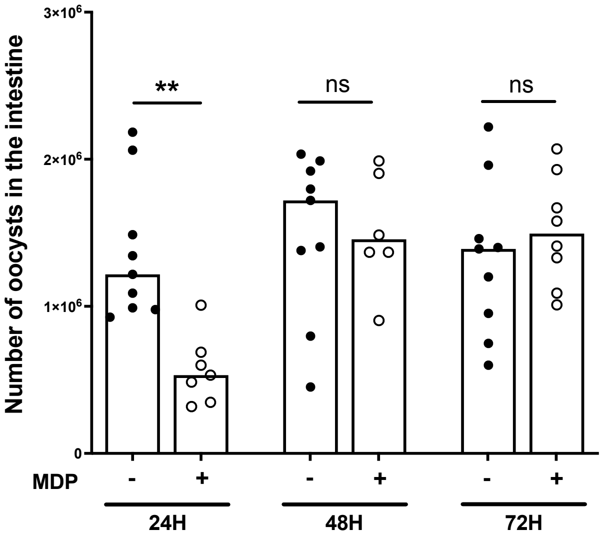

S1 Fig. Rapid and transient impact of NOD2 stimulation by MDP on *C. parvum* infection in neonatal mice. PND3 neonatal mice were orally infected with 5.10^5 oocysts of *C. parvum* and received 200µg of MDP (NOD2 ligand) or PBS (control group) by IP route. Parasite load in the intestine was evaluated 24H, 48H and 72H later (n = 7–9 mice for each group). Each point corresponds to one mouse, and bars represent the median of each group. Statistics are calculated by the Mann-Whitney test. ns>0.05, **P<0,01, ****P<0,0001. PND = postnatal days.

S2 Fig.

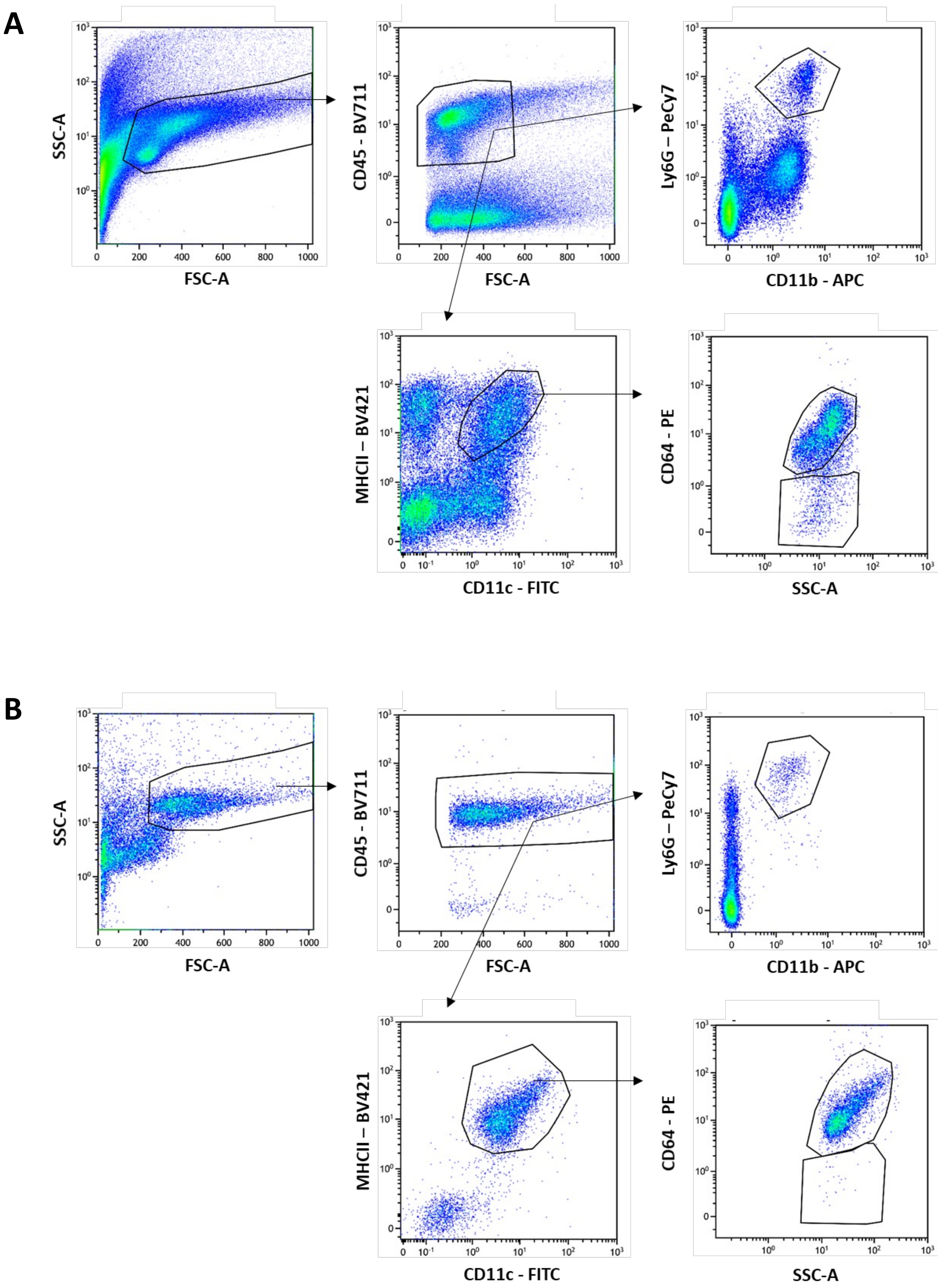

S2 Fig. Gating strategies for multicolor flow cytometry on lamina propria cells (A) or on IP cells (B). Neutrophils: CD45+ Ly6G+ CD11b+. Monocytes/Macrophages: CD45+ CMHII+ CD11c+ CD64+. Dendritic cells: CD45+ CMHII+ CD11c+ CD64-

S3 Fig.

Gene Name	Forward (5'-3')	Reverse (5'-3')
ANG4	CACAGCTAGACTCGTCCCCA	GGAGGAATCACAACCAGACCC
CCL20	CTTCCTTCCAGAGCTATTGTGG	TCATCCATTGGACAAGTCCACTG
CRS	ATGAAGAGACTTGTCCTCCTC	CTTCTTGAAGAGCAGAGCCTT
CRAMP	CCCAAGTCTGTGAGGTTCCG	AGGCAGGCCTACTACTCTGG
MMP7	TTTGATGGGCCAGGGAACACTCTA	ATGGGTGGCAGCAAACAGGAAGT
REG3β	TGGCTCCTACTGCTATGCCT	ACCAGTAAATTTGCAGACATAGGGC
REG3γ	CTCCTGCCTGATGCTCCTTTCT	CACCTCTGTTGGGTTCATAGCC
CXCL1	CGCTCGCTTCTCTGTGCAGC	GTGGCTATGACTTCGGTTTGG
CXCL2	GCTGCTGGCCACCAACCACC	TGAGAGTGGCTATGACTTCTG
HPRT	TGGATACAGGCCAGACTTTGTT	CAGATTCAACTTGCGCTCATC
IL-1β	CTTTGAAGAAGAGCCCATCCTCT	CATGGAGAATATCACTTGTTGGTTG
IL-6	GAGGATACCACTCCCAACAGACC	AAGTGCATCATCGTTGTTCATACA
IL-12p40	CTCACATCTGCTGCTCCACAA	GACGCCATTCCACATGTCACT
IL-13	CTTGCTTGCCTTGGTGGTCTCGC	GGTATCGGGGAGGCTGGAGA
IL-22	CAACTTCCAGCAGCCATACA	GTTGAGCACCTGCTTCATCA
PPIA	GCGGCAGGTCCATCTACG	GCCATCCAGCCATTCAGTC
S100A8	TCCTTTGTCAGCTCCGTCTTC	CTTCTCCAGTTCAGACGGCA
S100A9	GTGGAAGCACAGTTGGCAAC	TGGGTTGTTCTCATGCAGCT
TGF-β	GACCCCCACTGATACGCCT	GCTGAATCGAAAGCCCTGTA
TNF-α	ATGAGCACAGAAAGCATGATC	TACAGCCTTGTCACTCGAATT

S3 Fig. Primer sequences used for q-RT-PCR

S4 Fig. 24h

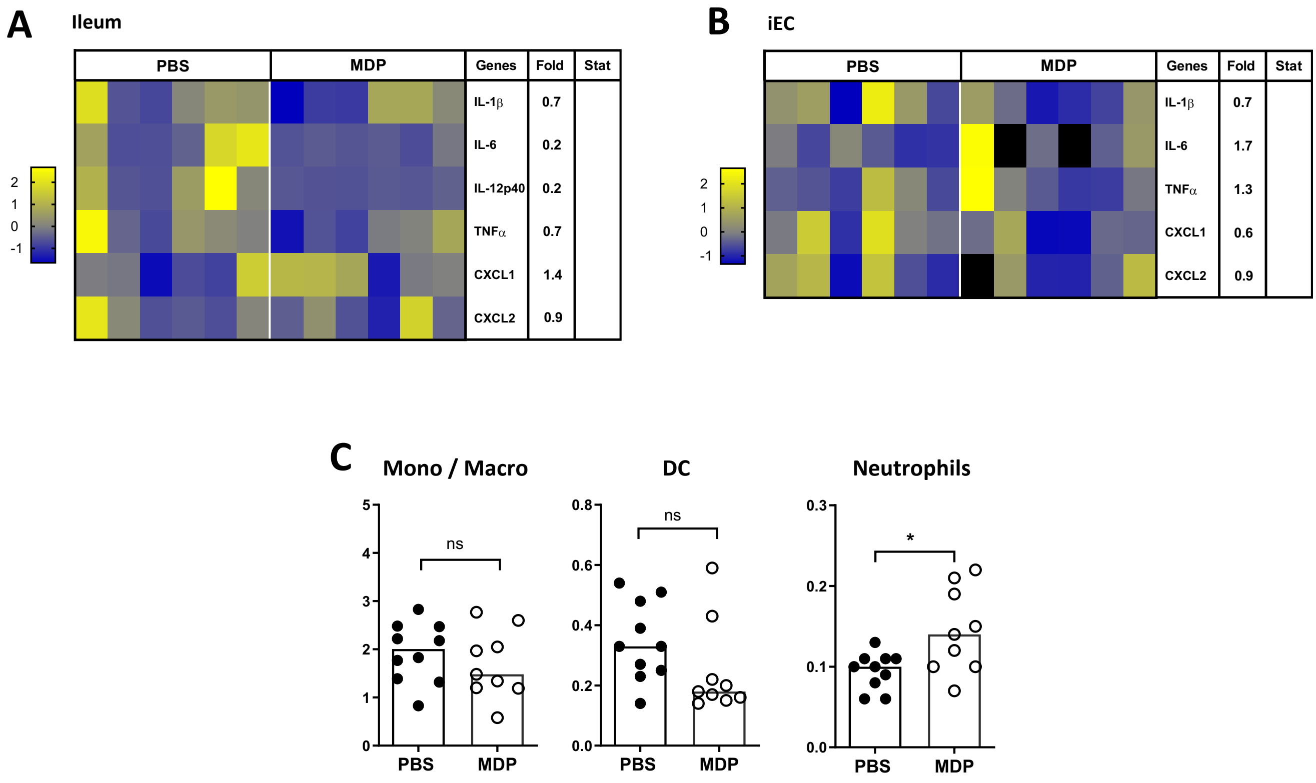

S4 Fig. MDP injection has an impact on neutrophils recruitment in the intestine of neonatal mice infected by *C. parvum* 24 h later PI. Neonatal mice were orally infected with 5.10^5 oocysts of *C. parvum* at 3-days-old and received an injection of MDP by IP route (200µg) 5 d.p.i.. 24 h after MDP injection, the intestine was sampled. (**A-B**) The levels of inflammatory gene expression were quantified by RT-qPCR in the ileum tissue (**A**) and in purified intestinal epithelial cell (IECs) (**B**). Heat maps are designed by z-score of the $2^{-\Delta ct}$ results for each gene and fold change of mRNA expression calculated as $2^{-\Delta ct}$ results of the MDP-group in comparison to the PBS-group (n = 6 mice for each group). (**C**) Cells from the intestinal *lamina propria* were collected and analyzed by flow cytometry. The percentage of monocytes/macrophages CD45+ CD11c+ MHCII+ CD64+, dendritic cells (DC) CD45+ CD11c+ MHCII+ CD64-, neutrophils CD45+ Ly6G+ CD11b+ was determined. (n = 9-10 mice for each group). Statistics are calculated by the Mann-Whitney test. ns >0.05, *P<0.05.

S5 Fig.

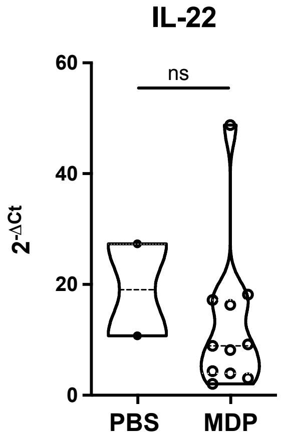

S5 Fig. **MDP injection does not change IL-22 expression in neutrophils from *C. parvum*-infected neonatal mice.** PND3 neonatal mice were orally infected with 5.10^5 oocysts of *C. parvum* and received 200µg of MDP by IP route at day 5 d.p.i.. 4 h after MDP injection, the intestine was sampled. Cells from the *lamina propria* of the distal small intestine were collected. Neutrophils were sorted, and the levels of IL-22 expression was quantified by RT-qPCR. Results are expressed as the $2^{-\Delta ct}$ compared between the MDP-group (n = 11 mice) and the PBS-group (n = 2, pool of 9 mice). Statistics are calculated by the Mann-Whitney test. ns>0.05.
